# Mesenteric Ischemia Caused by Heparin-induced Thrombocytopenia: A Case Report

**DOI:** 10.7759/cureus.3900

**Published:** 2019-01-16

**Authors:** Mohamed Ahmed, Saba Habis, Michael Samotowka, Ahmed Mahmoud, Michael Chin

**Affiliations:** 1 Surgery, Riverside Community Hospital, Riverside, USA; 2 Internal Medicine, Riverside Community Hospital, Riverside, USA; 3 Surgery, Jackson Memorial Hospital, Jacksonville, USA

**Keywords:** heparin-induced thrombocytopenia (hit), gunshot wound, mesenteric ischemia

## Abstract

Deep venous thrombosis (DVT) prophylaxis is the current standard of care in trauma patients. While most of the anticoagulation complications are obvious and can be promptly identified, heparin-induced thrombocytopenia (HIT) requires a high index of suspicion for early diagnosis to prevent a potentially fatal outcome. A 39-year-old female presented to our emergency room after sustaining a gunshot wound to her left flank and pelvis requiring a sigmoid colon and left fallopian tube and ovary resection with colostomy diversion. The patient did well in her early postoperative period; however, her condition rapidly deteriorated after that as a result of mesenteric ischemia caused by heparin-induced thrombocytopenia. After multiple visits to the operating room, the patient ended up with short bowel syndrome, necessitating a referral to a bowel transplant center. Our aim is to increase awareness of this potentially lethal complication with a mortality rate up to 30% of patients.

## Introduction

Heparin was introduced into clinical practice by McLean in 1916 with heparin-induced thrombocytopenia (HIT) reported more than 30 years later [1]. Rhodes identified the heparin-dependent antibody as the cause of HIT and thromboembolism [2]. The incidence of arterial thrombosis in HIT ranges between (3% - 10%), while venous thrombosis is much higher (17% - 55%) [3]. The “4 T's” scoring system is a pretest clinical scoring system and can be used as a guide to identify patients who are at high, intermediate, or low risk of developing HIT [4]. This case illustrates the significant morbidity of HIT and reviews the current management recommendations.

## Case presentation

A 39-year-old female was brought to our emergency room by a private vehicle after being shot. An evaluation revealed a wound at the lower left back and at the mons pubis. Exploratory laparotomy and resection of the sigmoid colon, left ovary, and fallopian tube with a colostomy was performed. Postoperative deep venous thrombosis prophylaxis in the form of enoxaparin, 30 mg every 12 hours, was given and then changed to heparin, 5,000 units subcutaneously every eight hours, due to worsening renal function. The patient did well until postoperative day 4 when she complained of increased abdominal pain out of proportion to the clinical findings. Her symptoms worsened the following day. A computed tomography (CT) scan of the abdomen and pelvis was obtained, the results of which were consistent with postoperative ileus and raising concerns for right colon ischemia.

**Figure 1:**
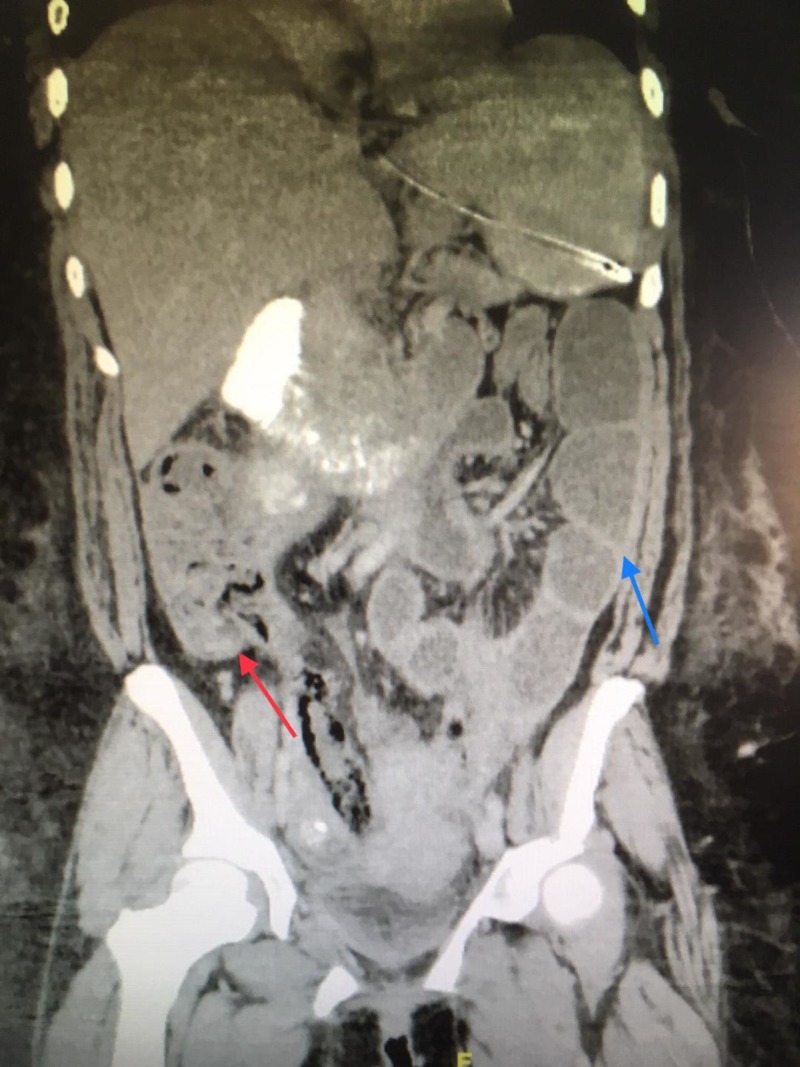
Computed tomography of the abdomen and pelvis Raising more concerns for ischemic bowel. Red arrow: thickened colon wall; blue arrow: thickened small bowel wall

The patient was managed conservatively, and bedside drainage of the superficial wound infection was done on postoperative day 6 with improvement in abdominal pain. On the following day, a repeat CT of the abdomen and pelvis raised more concerns for bowel ischemia; however, the patient refused reexploration (Figure 1).

A computed tomography angiogram (CTA) of the chest was obtained for worsening pulmonary symptoms on postoperative day 8 and was consistent with lower lobe pneumonia.

**Figure 2:**
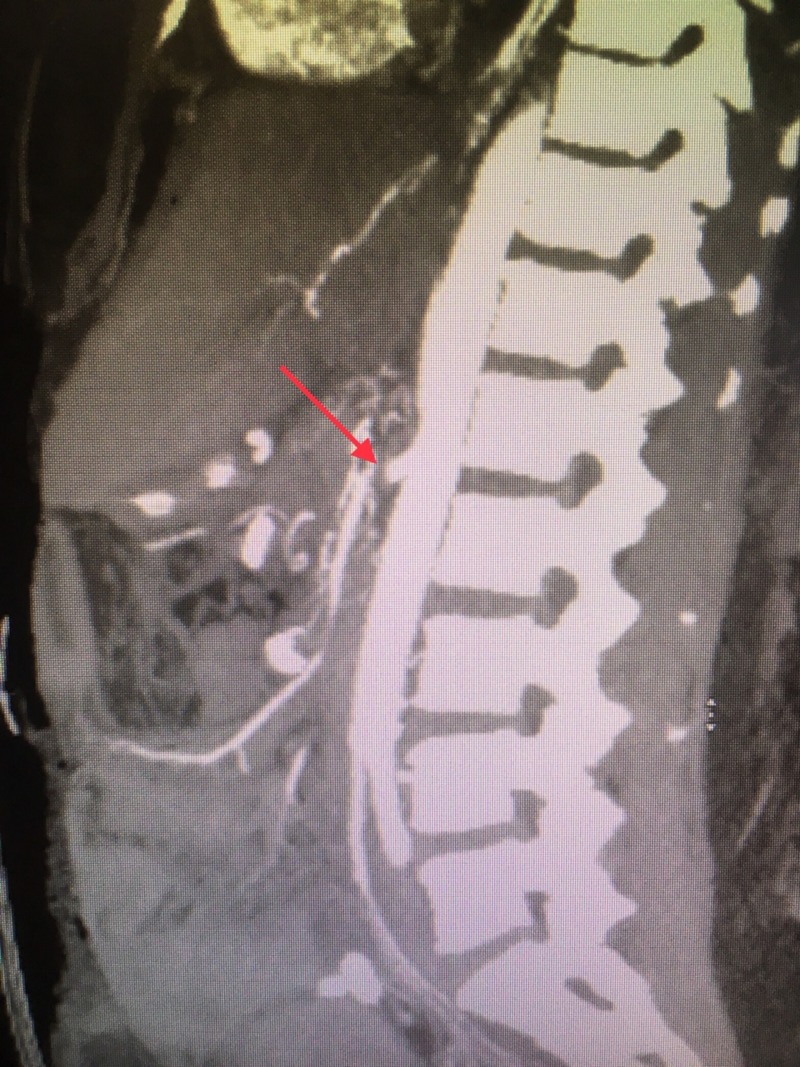
Computed tomography angiogram of the abdomen Superior mesenteric artery occlusion (red arrow)

Feculent drainage around the stoma was noticed on postoperative day 9, and an exploratory laparotomy was performed with resection of multiple small bowel ischemic loops, cholecystectomy, and right hemicolectomy. The rest of the small bowel appeared dusky and friable. After multiple visits to the operating room, she was left with only a few inches of the proximal jejunum. A CTA of the abdomen was done on postoperative day 13 from her initial surgery and was consistent with a superior mesenteric artery occlusion (Figure 2).

Admission platelet count was 281, down to 82 on postoperative day 11 when she tested positive for heparin-induced antibodies. The pre-test probability for HIT score was high (2 for thrombocytopenia, 1 for timing, 2 for thrombosis, 2 for no other cause = 7). The heparin was stopped and argatroban was used. The patient did survive her injury and was referred to a small bowel transplant center.

## Discussion

The incidence of HIT incidence is 0.1% - 5% in patients receiving heparin with 35% - 50% of those patients developing thrombosis. It should always be suspected in patients receiving heparin who develop a new onset thrombocytopenia with platelet counts are less than 150,000, or there is a drop of 50% or more in the platelet count, venous or arterial thrombosis, skin necrosis at the site of the injection, and if the patient develops acute systemic reactions after intravenous (IV) administration of heparin (fever, chills, tachycardia, hypertension, dyspnea, cardiopulmonary arrest). Antibody formation typically requires four or more days of exposure to heparin and presents with a dropping platelet count within five to 14 days. HIT is subdivided into two subtypes: HIT Type I (none immune and usually resolves spontaneously in few days) and HIT Type II which is immune-mediated (immunoglobulin G (IgG) antibody against heparin-platelet factor 4 (PF4) complex) resulting in excessive thrombin generation that leads to venous or arterial thrombosis [5].

Risk factors include surgery, the use of unfractionated heparin or low-molecular-weight heparin, heparin dose, female sex, and age above 40 [6-8]. The most common manifestation is thrombocytopenia. Venous thrombosis is more common (17% - 55%) than arterial thrombosis, manifesting (3% - 10%) as deep venous thromboses/pulmonary embolism, skin necrosis at the site of heparin, limb gangrene, and organ ischemia [3, 9-10]. Anaphylaxis that can be fatal has been reported [11].

Testing for the HIT antibody can be done with the use of the enzyme-linked immunosorbent assay (ELISA) or functional (serotonin release, heparin-induced platelet aggregation) assays. The former has a higher incidence of false positivity and the latter is more resource intensive and specific [12].

Once HIT is suspected, heparin or low-molecular-weight heparin (LMWH) should immediately be stopped. even prior to having the lab test results confirming the diagnosis, and a different DVT prophylaxis, such as argatroban or fondaparinux, should be started [13]. Warfarin is an anticoagulant but should not be used when you suspect HIT and actually needs to be discontinued due to increased bleeding risk when used in a patient with HIT. Adjunct therapy includes thrombectomy when feasible and debridement of any necrotic tissue, if possible [14].

## Conclusions

Heparin-induced thrombocytopenia complications can occur before thrombocytopenia is evident. The 4 T's score quantifies clinical findings and helps establish the pretest probability of HIT. A low index of suspicion should prompt stopping the drug and substituting alternative anticoagulants, such as argatroban or lepirudin. 
